# Interpretation of T‐wave inversion in physiological and pathological conditions: Current state and future perspectives

**DOI:** 10.1002/clc.23365

**Published:** 2020-04-07

**Authors:** Flavio D'Ascenzi, Francesca Anselmi, Paolo Emilio Adami, Antonio Pelliccia

**Affiliations:** ^1^ Department of Medical Biotechnologies, Division of Cardiology University of Siena Siena Italy; ^2^ Health and Science Department World Athletics Monaco Monaco; ^3^ Department of Exercise Human and Health Sciences, Foro Italico University of Rome Rome Italy; ^4^ Institute of Sports Medicine and Science Rome Italy

**Keywords:** athletes, athlete's heart, cardiomyopathy, death, electrocardiography, negative T‐waves, sports cardiology, sudden cardiac

## Abstract

The presence of T‐wave inversion (TWI) at 12‐lead electrocardiogram (ECG) in competitive athletes is one of the major diagnostic challenges for sports physicians and consulting cardiologists. Indeed, while the presence of TWI may be associated with some benign conditions and it may be occasionally seen in healthy athletes presenting signs of cardiac remodeling, it may also represent an early sign of an underlying, concealed structural heart disease or life‐threatening arrhythmogenic cardiomyopathies, which may be responsible for exercise‐related sudden cardiac death (SCD). The interpretation of TWI in athletes is complex and the inherent implications for the clinical practice represent a conundrum for physicians. Accordingly, the detection of TWI should be viewed as a potential red flag on the ECG of young and apparently healthy athletes and warrants further investigations because it may represent the initial expression of cardiomyopathies that may not be evident until many years later and that may ultimately be associated with adverse outcomes. The aim of this review is, therefore, to report an update of the literature on TWI in athletes, with a specific focus on the interpretation and management.

## 1 INTRODUCTION

T‐wave inversion (TWI) is defined as negative T‐wave of ≥1 mm in depth in two or more contiguous leads, with exclusion of leads aVR, III, and V1.^1^ The presence of TWI at 12‐lead electrocardiogram (ECG) in competitive athletes is one of the major diagnostic challenges for sports physicians and consulting cardiologists.

The interpretation of TWI in athletes is complex and the inherent implications for the clinical practice represent a conundrum for physicians. TWI may be occasionally seen in healthy athletes presenting signs of cardiac remodeling, such as left ventricular (LV) hypertrophy, atrial dilation, increase in ventricular cavity size, which may occasionally overlap with those of life‐threatening arrhythmogenic cardiomyopathies. In some cases, the identification of TWI should be viewed as a red flag on the ECG of young and apparently healthy athletes and warrants further investigations, having in mind that it may represent the initial expression of cardiomyopathies that may not be evident until many years later and that may ultimately be associated with adverse outcomes.^2^ The aim of this review is, therefore, to report an update of the literature on TWI in athletes, with a specific focus on the interpretation and management, including the advice for eligibility and/or disqualification from competitions.

For a proper interpretation of TWI patterns in athletes, it is crucial to consider primarily the TWI localization, which may be helpful to identify specific cardiac pathologies, in association with the family and personal history and the clinical correlates of the TWI.

## 2 ANTERIOR TWI

Anterior TWI is defined as negative T wave in precordial leads exceeding V1. It should be reminded that TWI confined in V1‐V2 is common finding in the general population and may be observed also in endurance athletes (14%‐28%).^3^, ^4^ As a potential explanation of the presence of TWI in V1 and V2, a recent cardiac magnetic resonance (CMR) study demonstrated that this pattern may reflect a lateral displacement of the right ventricle, rather than a true right ventricular dilatation.^5^ A study on a large population of 14  000 subjects (age range: 16‐35 years), including 2958 athletes and 11  688 non‐athletes, reported a prevalence of anterior TWI in 2.3%, being more common in females and in most cases confined to leads V1 and V2.^6^ None of these subjects was diagnosed with a cardiomyopathy after a comprehensive evaluation, suggesting that this pattern is benign, in the context of the low‐risk asymptomatic population. Concordantly, the Seattle and the new International Criteria recommend to only investigate non‐black athletes with anterior TWI beyond V2, in absence of other clinical or electrical signs of arrhythmogenic cardiomyopathy (AC).^7^, ^8^

Anterior TWI in precordial leads exceeding V2 may also be a physiological variant in adolescent athletes of ≤14 years, or in black (Afro‐Caribbean) athletes (both adolescent and young adults) when preceded by J‐point elevation and convex ST‐segment elevation, but also in some Caucasian endurance athletes with a persistent “juvenile pattern”^4^, ^9^, ^10^, ^11^ (see section: TWI in specific populations).

**FIGURE 1:**
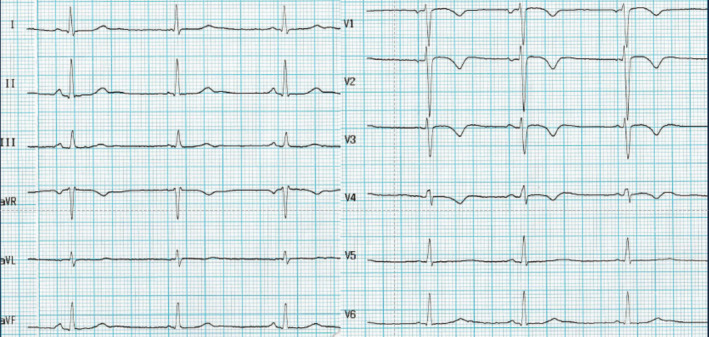
Electrocardiogram of a 24‐year‐old female tennis player with T‐wave inversion from V1 to V4 at the preparticipation evaluation. The subsequent clinical investigations confirmed a definitive diagnosis of arrhythmogenic cardiomyopathy

In the clinical setting, anterior TWI is a recognized hallmark of AC (see Figure 1) and (more rarely) of hypertrophic cardiomyopathy (HCM). This pattern is observed in 2% to 4% of patients with HCM, but it may be present in as many as 80% of patients with AC experiencing adverse events.^12^, ^13^ Currently, anterior TWI is a major criterion for the diagnosis of AC.^14^ Specifically, inverted T waves in right precordial leads (V1, V2, and V3) or beyond, in individuals >14 years of age (in the absence of complete right bundle‐branch block QRS≥120 ms) constitute a *major* diagnostic criterion for AC, while inverted T waves in leads V1 and V2 in individuals >14 years of age (in the absence of complete right bundle‐branch block) is considered a *minor* criterion.^14^ Conversely, in the presence of complete right bundle‐branch block, only anterior TWI beyond V3 could be considered a minor criterion.^14^

Zaidi et al investigated the accuracy of these ECG criteria for diagnosis of AC when applied to athletes^15^ and demonstrated that isolated anterior TWI do not discriminate between patient with AC and healthy athletes. ECG features associated with physiological remodeling were the presence of J‐point elevation, early repolarization, biphasic TWIs, and voltage criteria for left and/or right ventricular hypertrophy.^15^ On the other side, ECG markers more commonly associated with AC included TWI preceded by an isoelectric or a depressed ST‐segment and the presence of low QRS voltages. Markers in favor of the diagnosis of AC included also symptoms, like syncope, presence of Q waves or precordial QRS voltages <1.8 mV, three abnormal SAECG parameters, delayed gadolinium enhancement identified by CMR, RV ejection fraction ≤45%, or wall motion abnormalities, and presence of premature ventricular beats.^15^, ^16^, ^17^, ^18^

Calore et al compared the ECG pattern in 80 healthy athletes with anterior TWI with 95 patients affected by HCM and 58 patients by AC.^10^ In this study, J‐point elevation <1 mm in the anterior leads showing TWI and TWI extending beyond V4 remained independent predictors for the diagnosis of either AC or HCM. On the other hand, in athletes with anterior TWI, the combination of J‐point elevation ≥1 mm and TWI not extending over V3 excluded a cardiomyopathy, either AC or HCM (100% of sensitivity and 55% of specificity).^10^ Conversely, a depressed ST‐segment was considered an electrical marker of cardiac pathology.^6^ These results emphasize the importance of assessing the J termination (Jt) and ST‐segment preceding TWI, as further markers to differentiate between physiological cardiac adaptation and a cardiomyopathy.^10^ However, there is still a concern regarding the accuracy of J‐point elevation as a discriminant of pathology in athletes with anterior TWI. Finocchiaro et al. reported that a J‐point <0.1 mV preceding anterior TWI is not specific of AC, because it is present in the majority of healthy individuals, including athletes.^19^ Similarly, Brosnan et al studied the electrocardiographic features differentiating AC from athlete's heart and found that J‐point elevation had poor specificity (27%) and accuracy (60%) in identifying healthy athletes, being a poor discriminator of health vs disease condition.^20^

In summary, a minimal or absent J‐point elevation (<1 mm) or a coexistent depressed ST‐segment preceding TWI could suggest the presence of an underlying cardiomyopathy. However, this finding may be observed also in healthy people, so additional ECG alterations suspected for AC, such as low limb lead voltages, prolonged S‐wave upstroke, ventricular ectopy with uncommon morphology, and epsilon waves should be ascertained. On the contrary, findings such as distinct J‐point elevation, ST‐segment elevation, or biphasic T waves more likely represent athlete's heart. Notably, among healthy individuals with anterior TWI, ST‐segment elevation was more common in men than in women regardless of athletic status and, in women with anterior TWI, the ST‐segment was most commonly isoelectric,^6^ Finally, a comprehensive evaluation including information on family history and the presence of symptoms provides additional help in differentiating between pathological vs physiological pattern.^10^, ^14^

## 3 LATERAL TWI

The presence of TWI in leads I and AVL, V5 and/or V6 (only one lead of TWI required in V5 or V6) is defined as lateral TWI, while the presence of TWI in leads II and aVF, V5‐V6, I and AVL is defined as infero‐lateral TWI.^7^

**FIGURE 2:**
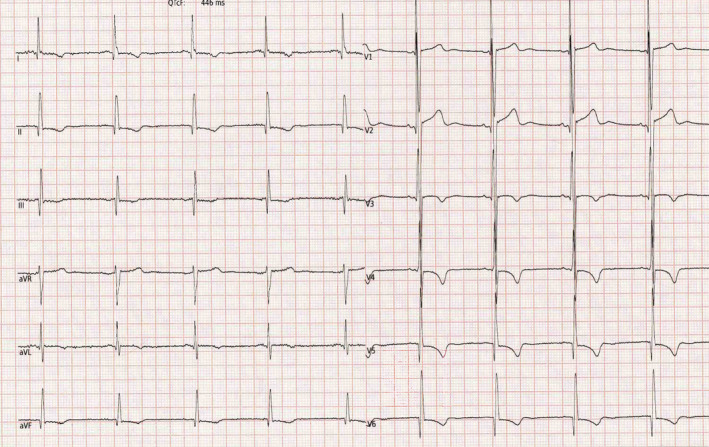
Electrocardiogram of a 30‐year‐old male basketball player with T‐wave inversion in the lateral and inferior leads with concomitant Q waves. The subsequent clinical investigations confirmed a definitive diagnosis of hypertrophic cardiomyopathy

The prevalence of lateral TWI in athletes varies between 0.3% and 1.5%^21^, ^22^ and it seems to be 10 times higher in black than white athletes.^23^ The presence of lateral and inferolateral TWI is associated with the existence of a cardiomyopathy in a large number of athletes^2^, ^9^, ^11^, ^22^, ^24^ (see Figure 2). Accordingly, these patterns of TWI should always raise the suspicion of an underlining heart disease and should prompt a comprehensive investigation in order to exclude a life‐threatening cardiac condition.^7^ Even if cardiac pathology is not diagnosed at the first evaluation, a clinical follow‐up including cardiac imaging testing, with serial (annual during adolescence and young adulthood) evaluations, is recommended in order to timely identify the development of a cardiomyopathy.^7^ From a database of 12  550 athletes referred for pre‐participation screening, Pelliccia et al identified 81 with diffusely distributed and deeply inverted T waves (≥2 mm in at least three leads) who had no apparent cardiac disease at initial evaluation and followed them up for a period of 9 (and up to 27) years.^2^ A cardiomyopathy developed in five athletes (6%; 1 AC, 3 HCM, 1 DCM) during the period of follow‐up and other cardiovascular disorders developed in six athletes (7%; hypertension, atherosclerotic coronary artery disease, myocarditis, and supraventricular tachycardia).^2^ All these athletes exhibited lateral or inferolateral TWI already at initial evaluation.

Schnell et al performed an investigation on 155 asymptomatic athletes who exhibited deep TWI in two or more leads, of which 137 (88%) exhibited lateral deep TWI.^24^ Evidence of cardiomyopathies was present in 41% of subjects with lateral TWI, with HCM being the predominant diagnosis. In this study, CMR detected 24 cases of disease in athletes whose echocardiogram was normal or inconclusive, with HCM being the most common diagnosis, followed by AC.^24^

Sheikh et al investigated asymptomatic black and white athletes with TWI and observed that 21% of athletes were diagnosed with cardiac disease, with a prevalence higher in white than black athletes (30% vs 12%, *P* = .027).^23^ Almost all athletes with a clinical diagnosis of cardiomyopathy exhibited lateral TWI.

Based on this experience, it is sound to conclude that all athletes with lateral and/or infero‐lateral TWI should undergo further evaluation. Even if echocardiography shows no structural heart disease, CMR should be preferentially performed. The CMR provides superior results in the evaluation of HCM, especially for left ventricular apex, and offers the unique possibility to characterize myocardial tissue, identifying the presence of myocardial fibrosis. Exercise testing and Holter ECG monitoring may also be useful, when ventricular arrhythmias with uncommon morphology appear with exercise, to support diagnosis of the cardiomyopathy.^25^

## 4 INFERIOR TWI

The presence of TWI in leads II and AVF is defined as inferior TWI. The clinical significance of isolated inferior TWI is still uncertain. Inferior TWI has been reported in a certain proportion of normal individuals including healthy athletes, but it may be present in subjects affected by a cardiomyopathy^7^ *(ref. abstract, see references list). In patients with AC, the prevalence of inferior TWI can reach the 31%, while in healthy athletes, the prevalence is lower (about 3%).^20^

Inferior TWI may also be observed in valvular heart disease, first of all in mitral valve prolapse (MVP).^26^ Although MVP is relatively common in athletes and usually characterized by a benign course,^27^ it may rarely present with ominous ventricular arrhythmias eventually leading to sudden cardiac death (SCD), even in the absence of relevant hemodynamic impairment. Specifically, Basso et al examined 46 cases of SCD with MVP being the only pathological condition linked to SCD. Of these, 10 of 12 patients (mostly, adult females) with available ECGs had inverted T waves in inferior leads.^26^ Furthermore, MVP patients at risk of SCD had bi‐leaflet involvement of the mitral valve, mid‐systolic click at auscultation, T‐wave abnormalities on inferior leads, RBBB‐type or polymorphic ventricular arrhythmias and evidence of myocardial scarring at CMR, localized in the posterior papillary muscle and the infero‐basal LV free wall, confirming the site of origin of RBBB‐type ventricular arrhythmias. Therefore, this finding suggests that athletes with clear evidence of MVP and T‐wave abnormalities on inferior leads should undergo a clinical evaluation including at least echocardiography, a 24‐hour 12‐lead Holter ECG monitoring and eventually an exercise testing. In presence of MVP with TWI and substantial arrhythmic burden, CMR is advised.

In summary, the presence of inferior TWI is relatively common in young athletes and its clinical significance is uncertain when present in isolation. Further evaluation should be considered in the presence of symptoms, additional ECG anomalies or abnormal morphologic cardiac findings.^7^

## 5 TWI IN SPECIFIC POPULATIONS

### 5.1 Children

The interpretation of TWI on the 12‐lead resting ECG in children can be challenging because of the presence of the peculiar electrical remodeling observed during the body growth. Indeed, from birth to adolescence, dynamic repolarization changes are normally seen in the anterior leads and anterior TWI in children reflects the right ventricular dominance.^6^ The switch from the right to the left electrical predominance results in a gradual reversal of T‐wave polarity. After puberty, the T wave presents the same characteristics of the adults and is usually inverted in only lead V1, and upright in leads V2‐V6.^12^ However, anterior TWI may persist after puberty and young athletes often exhibit TWI in the right precordial leads as a representation of normal juvenile ECG pattern but similar to the repolarization abnormalities observed in cardiomyopathies.

According to the current Task Force criteria for the diagnosis of AC, the age threshold is 14 years after that anterior TWI cannot be classified as normal, and the presence of inverted T waves in leads V1 and V2 in individuals >14 years represents a minor criterion for the diagnosis of AC.^14^

In a recent cross‐sectional study aimed to derive normal ECG values in children and adolescent engaged in non‐competitive sports, Molinari et al found that the presence of TWI decreased with age, from 55% to 60% at 3 years of age to 8% to10% at 14 years of age.^28^ In the study by Migliore et al, in a large population of children with a mean age of 13.9 ± 2.2 years (range 8‐18 years), the prevalence of TWI was 5.7%.^12^ The prevalence decreases as athletes progress from childhood to adolescence and young adulthood, as also demonstrated by Sharma et al who reported a prevalence of TWI in V2‐V3 up to 4% in a population of 1000 junior elite athletes with a mean age of 15.7 years.^29^ Similarly, Papadakis et al found a prevalence of 4% in adolescent athletes with a mean age 16 ± 1.7 years (range 14‐18 years) and showed that TWI extending beyond V2 was extremely rare (0.8%).^21^ In a selected cohort of 247 adolescent (mean age, 16 years) elite athletes undergoing ECG screening before the Youth Olympic Games, Adami et al observed TWI in 23 (9.3%) of the overall group; majority were localized in anterior leads (V1‐V3) and not associated with cardiac abnormalities; the only case with underlying cardiac disease showed TWI in infero‐lateral leads.^30^

A recent longitudinal study on 2227 children, pre‐pubertal and younger in comparison with the previous cited studies (mean age 12.3 ± 2.0 years) practicing sport, demonstrates a high prevalence of TWI, up to 16%.^9^ This longitudinal study confirmed that anterior TWI become positive in the vast majority of the children (94%) after a 4‐year follow‐up, with only 6% of children still exhibiting anterior TWI, in absence of family history, symptoms or relevant clinical findings. In this population, anterior TWI becomes positive by the age of 14 years in most of the children. Conversely, TWI in the infero‐lateral leads was rare (only 3% of children), persists after puberty and was also associated with structural heart disease in one case.^9^

In conclusion, an accurate differentiation between pathological and physiological ECG patterns is crucial in children with TWI, frequently presenting dynamic changes in the anterior leads, related to age and development. In this age group (such as in adults), TWI in the infero‐lateral leads should undergo further investigations to identify potential cardiomyopathies.^21^, ^31^ Conversely, in absence of symptoms and/or family history for SCD or cardiomyopathies, anterior TWI up to V3 in children ≤14 should not under further investigations and a yearly follow‐up is suggested until the positivization of anterior TWI. Conversely, when anterior TWI persists after the age of 14 years, further investigations including—but, depending on the case, not limited to—echocardiography should be performed in order to exclude the presence of structural heart disease. Similarly, the current international criteria for ECG interpretation in competitive athletes define anterior TWI from V1 to V3 as a normal ECG finding in children when the age is <16 years.^7^ As stated earlier, it is difficult to define a clear age cutoff and the decision to proceed with further investigations should take into account also clinical, demographic, and biological data.

### 5.2 Black athletes

**FIGURE 3:**
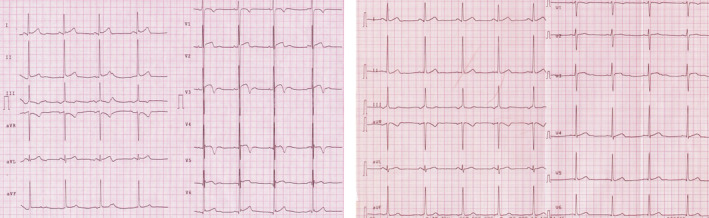
Anterior T‐wave inversion (TWI) in a 28‐year‐old male black athlete practicing basketball. On the left, the ECG showed negative T waves from V1 to V4 preceded by J‐point elevation and convex ST‐segment elevation; the electrocardiogram was collected at peak training. On the right, the electrocardiogram of the same athlete recorded after the period of low training regimen: a complete positivization of anterior TWI is observed after detraining in this subject, supporting the physiological interpretation of these data. ECG, electrocardiogram

Black athletes may present peculiar ECG patterns that differ from those observed in non‐black athletes. A recent review reported that about 10% to 30% of black African/Afro‐Caribbean athletes had abnormal ECG, with R/S voltage criteria for LV hypertrophy in 60% to 89% and ST‐segment elevation and TWI being common in this population.^32^ While lateral and inferior‐lateral TWI are universally recognized as abnormal, the international recommendations for the interpretation of ECG in athletes classify as normal the presence of anterior TWI in V1‐V4 when preceded by Jt and/or ST‐segment elevation in black athletes.^7^ Here again, observation of the J‐point and the preceding ST‐segment may help differentiate between physiological changes and cardiomyopathy (see Figure 3).

A comparison between anterior TWI in groups of black and white healthy athletes, with patients with HCM and AC, demonstrated that both in white and black athletes the presence of TWI in leads V1 to V4 preceded by J‐point elevation ≥1 mm excluded a cardiomyopathy (100% negative predictive value).^10^ A recent study by McClean et al investigated the prevalence and distribution of TWI in an adolescent and young population of Arab and black athletes (aged 11‐18 years) and reported that 15.8% presented anterior TWI, in the majority of cases confined to leads V1 to V3.^33^ In this study, the prevalence of anterior TWI and of Jt elevation was higher in Arabic athletes aged <16 years and in black athletes. Of notice, in three black athletes, a cardiac disease was diagnosed; in these cases, TWI in leads V1 to V4 was associated with other abnormal features, such as lateral TWI, Q waves, or wide QRS. Therefore, the interpretation of ECG in black athletes may be challenging, but the knowledge of physiological ECG variants in this ethnicity may useful to reduce the burden of false‐positive ECGs and to distinguish between physiological and pathological remodeling.

In summary, anterior TWI in black athletes should be considered a common ECG finding in presence of a J‐point elevation and convex ST‐segment elevation followed by TWI in V2‐V4, as recommended.^7^ Conversely, when anterior TWI is associated with other ECG anomalies or when the characteristics of anterior TWI does not suggest a benign pattern of repolarization (eg, TWI preceded by ST‐segment depression of deep TWI), further investigations are recommended.

### 5.3 Implications for sport eligibility

**FIGURE 4:**
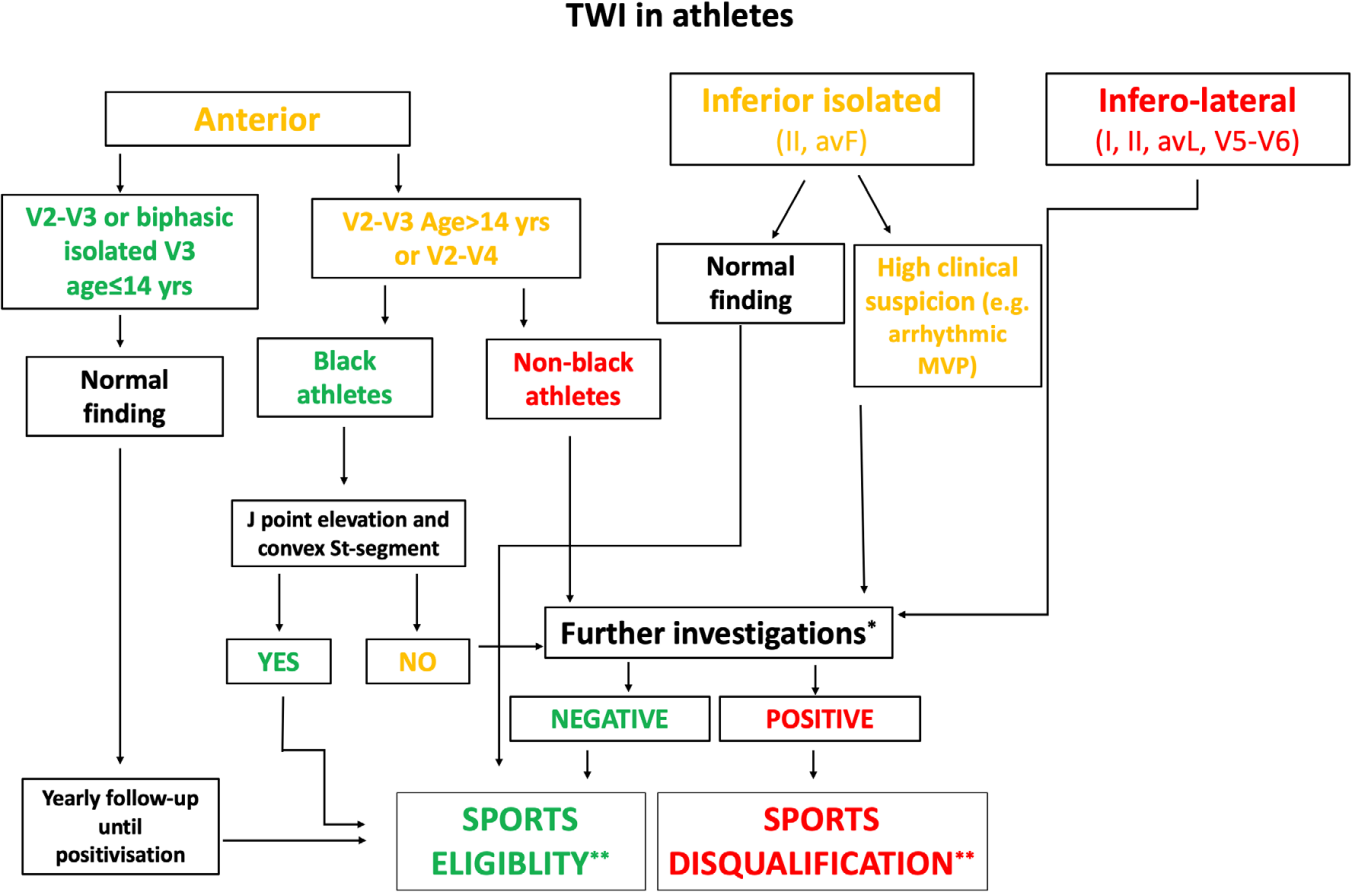
Proposed algorithm for evaluation of athletes with T‐wave inversion. * Further investigations include—but are not limited to—echocardiography, stress testing, 12‐lead Holter ECG and cardiac magnetic resonance. The indication to these diagnostic tests is based on a clinical decision and takes into account the personal and family history of the athlete as well as the results of the previous examinations; **Beyond sports eligibility or disqualification, a periodical follow‐up is usually needed in most of these cases, with a timeline based on the clinical characteristics of the athlete. Sports eligibility and disqualification should be guided by the recent position statement of the Sport Cardiology Section of the European Association of Preventive Cardiology.^34^ ECG, electrocardiogram

The presence of TWI is not, per se, a diagnosis of a cardiomyopathy. Therefore, in the presence of TWI, the physician should prompt the investigations aimed to exclude, or confirm, the presence of an underlying pathologic condition, as depicted in the flowchart (Figure 4).

Sport eligibility, in athletes with TWI, must be established according to the presence of the concomitant cardiac condition, and the sport participated, as suggested by the current ESC recommendations.^34^

It is of notice that, in most instances, a markedly abnormal ECG may not reveal any feature of cardiomyopathy. In this case, the athlete should not be considered as affected by a cardiac disease and therefore, no restrictions in his/her lifestyle, including participation in competitive sport should be advocated. Nevertheless, considering the potential for developing a cardiomyopathy later in life, regular follow‐up is highly recommended (annually, during adolescence, and young adulthood).^2^ Indeed, while cardiac imaging can be negative at the first clinical evaluations in children/adolescents with suspected TWI, a pathological LV hypertrophy can be detected later in the life in athletes with TWI and a strict clinical follow‐up is recommended for this purpose. Athletes should also be educated with respect to the identification of incident cardiac symptoms as a red flag requiring re‐evaluation.

## 6 FUTURE PERSPECTIVES

The interpretation of TWI in athletes and their clinical management can be sometimes challenging. Although several studies have been conducted with the aim of interpreting TWI in this specific population, knowledge gaps still exist. In particular, few data are available on the clinical significance of isolated TWI in the inferior leads. Furthermore, despite the large number of young subjects practicing sport, the literature is limited also in the specific setting of children engaged in competitions which exhibit dynamic electrocardiographic changes—and particularly in the anterior precordial leads—with a different interpretation in comparison with adults. Finally, the clinical course of athletes with abnormal TWI and negative/inconclusive cardiac imaging is another area of interest with limited data currently available. Further research is needed in these different settings in order to help physicians managing athletes with TWI.
